# Supplementary material for: Partner perceptions during brief online interactions shape partner selection and cooperation
Source: PLoS One. 2025 Apr 9;20(4):e0318137. doi: 10.1371/journal.pone.0318137 (PMC11981216; doi:10.1371/journal.pone.0318137)
Supplement: S1 File — (DOCX) [file pone.0318137.s001.docx]

**Supplementary Material: Partner Perceptions During Brief Online Interactions**

**Shape Partner Selection and Cooperation**

# **Supplementary Note 1: Methods: Participant Selection**

Participants were recruited from the UK via an online recruiting platform Prolific (h[ttp://www.prolific.co).](http://www.prolific.co/) Only participants who fulfilled the selection criteria were invited for the second part of the study: 1) had a working web-camera and headphones, 2) had their own PC or laptop, 3) had sufficient bandwidth (checked with an online self-test during the first part of the study), 4) were living in the UK at the time of the data collection. In total. 297 participants participated in both parts of the study and were thus included in further analysis. Participants who did not participate in all stages of the interaction part (i.e., dropped during the second part of the study due to connectivity problems or technical difficulties) and had missing values in the self-reported data were removed. Furthermore, due to the round-robin experimental design we had more dyads and observations than participants, as each participant provided evaluation for each conversation/photograph. The sessions differed in the amount of completeness (n_photograph_ = 1383, n_conversation_ = 1385; n_coordination_ = 1326; n_collaboration_ = 1149). Thus, to keep the same observations in all sessions, we decided to keep observations that happened only in all four sessions and had conversations longer than 2 minutes. The decision for using the 2-minute threshold was motivated by the study design choice, where participant could hang up after 2 minutes. Meaning, that even if the conversation was not 3 minutes long (as originally designed), 2 minutes conversations were still treated as valid. This resulted in reducing the number of observations from 1136 to 1080 coming from 540 dyads. The majority of participants (76.81 %) were originally from the UK, while 13.97 % participants were from other parts of Europe (i.e. Portugal, Spain, Norway), and a minority (9.06 %) of participants were from other continents (e.g., Africa, Australia, Asia). Nevertheless, all participants were living in the UK at the time of the study.

# **Supplementary Note 2: Experimental Tasks: Description and Instructions**

## Joint Trust Task

Joint Trust task was an adjusted version of the original Trust Task. (Berg, Dickhaut & McCabe, 1995). Each participant was given an initial endowment of 10 MU, where each MU was worth 0.50 pounds. Participants decided how to distribute the MU between themselves and their current interaction partner. The task was repeated one time for each dyad within the batch. Thus, irrespective of their partner selection each participant played the Joint Trust Task once with each participant within their batch. MU’s each participant decided to give to the other person were multiplied during the transaction and were worth more for their partner than for them (i.e., MU after transaction were multiplied by 1.20). Participant were free to choose how much MU to give to the other person (0-10MU). The maximum amount each participant could earn was 22MU (11 euros). The maximum amount was earned if the participant decided to keep all the MU for themselves and their partner gave them all their MUs. The task is a representation of a social dilemma, as there is a conflict of interest and incentive to exploit the partner in the task. However, this option is risky, and if both participants think of each other’s benefit the best mutual outcome is when both of them decide to allocate the entire endowment to their partner (i.e. 6 euros)., relative to both of them keeping the endowment to themselves (i.e., 5 euros). Compared to the Joint Competence Task (see below), this task is highly interdependent where individuals face a conflict of interest. Each person has an incentive to exploit the other person, as such this task affords for each person’s concern for their partner’s benefit (i.e. warmth, trustworthiness), while partner’s competence is less afforded to impact outcomes in this task.

## Joint Competence Task

The Joint Competence Task was designed to be an interdependent task whereby each participant could ensure a mutual benefit from themselves and their partner. In this task, each participant as given an initial endowment which was proportional to their performance in the UCMRT test. UCMRT test was the intelligence task individuals solved during the first part of the study. Each point on the UCMRT test was worth 0.50 pounds. Due to the length of the UCMRT task (23 problems), individuals were able to have 11.5 euros as their initial endowment. At the beginning of the task, the participant’s endowment was jointed with the partner’s endowment and put into the shared pool. This calculation was done for every pairing doing the task. The money in the share pool could be won by solving one additional problem taken from the new and unseen version of the UCMRT task. Each pair solved a different problem. All problem were calculated for difficulty and were equal on the difficulty level (level of difficulty = 0.70). Each participant was solving the problem independently of their partner. However, the performance affected both of their earnings. Specifically, there were three possible outcomes given their performance. Firstly, if both participants provided the correct response on the problem, then each participant would receive half of the money from the shared pool. Second potential outcome was if one participant did not provide the correct response, then each participant would receive one fourth of the money from the shared pool. Finally, if both participants did not solve the task correctly, they lose the money from the shared pool. In this task, people are interdependent with corresponding interests. However, each person earns more money when both solve the task correctly. Hence, this task affords for partner competence to influence outcomes of the task, while partner’s warmth is relatively less relevant for the task outcomes.

# **Supplementary Note 3: List of OpenFace and OpenSmile Feature**

**Table 1**

*List of features extracted with off-the-shelf algorithms OpenFace and OpenSmile (see also Eyben, Wollmer & Schuller, 2010)*

| OpenFace | Action units (AU01, AU02, AU04, AU05, AU06, AU07, AU09, AU10, AU12, AU15, AU17, AU20, AU25, AU26, AU45) |
| --- | --- |
|  | Eye gaze angle and direction |
| OpenSmile | Waveform (Zero-Crossings, Extremes, DS) |
|  | Signal energy (Root-mean square & logarithmic) |
|  | Loudness (Intensity & approximate loudness) |
|  | FFT spectrum (Phase, magnitude) |
|  | ACF & Cepstrum (Autocorrelation and Cepstrum) |
|  | Mel spectrum |
|  | Cepstral (Cepstral features - MFCC, PLP-CC) |
|  | Pitch (fundamental frequency) & Probability of Voicing |
|  | Voice Quality (HNR, Jitter and Shimmer) |
|  | LPC (LPC coefficinets, Line Spectral Pairs (LSP) |
|  | Auditory (Auditory spectra & PLP coefficients) |
|  | Formants (Centre frequencies and bandwidths) |
|  | Spectral (centroid, flux, entropy, max./min) |
|  | Tonal (CHROMA, CENS, CHROMA -based features) |

# **Supplementary Note 4: Distribution of Self-reports from the Intake Session**

**Fig 1.***
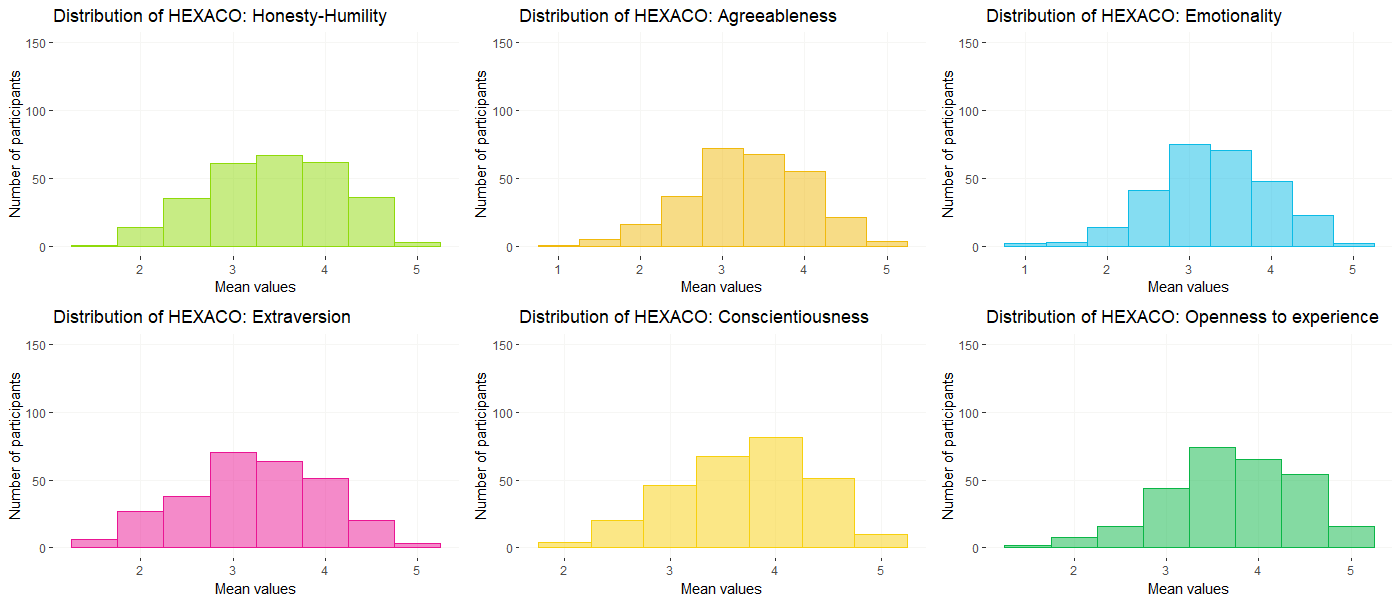
* *Distributions of HEXACO-60 self-reports on each personality facet of the scale*

**
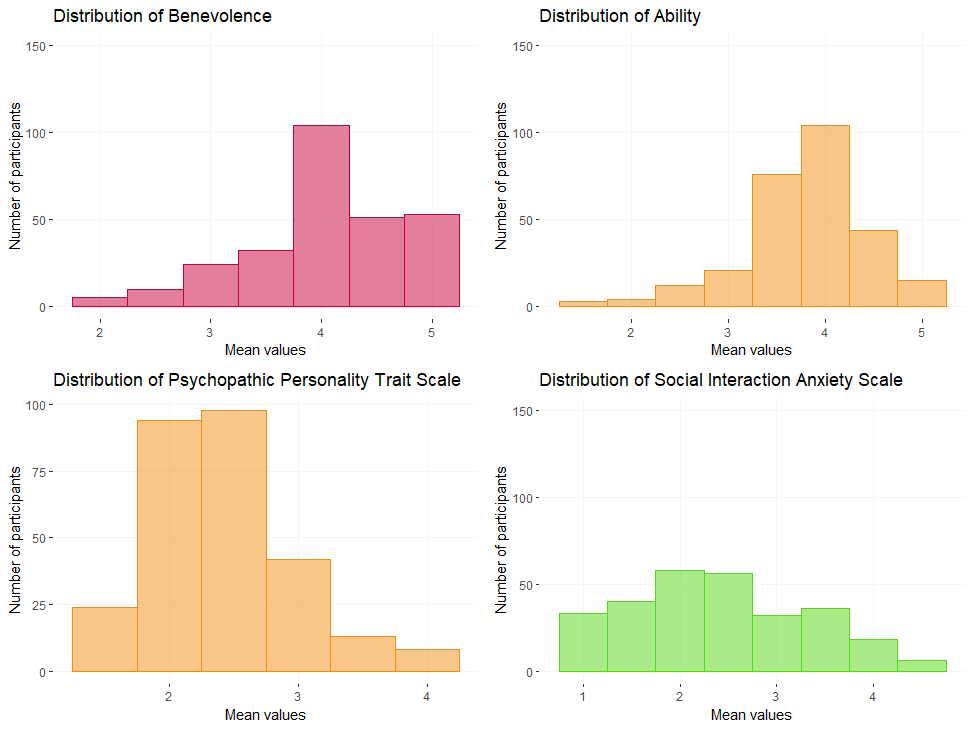
Fig 2.** *Distribution of two ABI model scales: Benevolence and Ability, Psychopathy and Social Anxiety*

# **Supplementary Note 5: Descriptive Statistics of Evaluations**

**Table 2**

| Variables | Overall | | | | | | Selected Partners | | | | | | | Unselected Partners | | | | | | | |
| --- | --- | --- | --- | --- | --- | --- | --- | --- | --- | --- | --- | --- | --- | --- | --- | --- | --- | --- | --- | --- | --- |
|  | Photographs | | | Conversations | | | Photographs (n = 759) | | | Conversations (n = 771) | | | | Photographs (n = 321) | | | | Conversations (n = 309) | | | |
|  | *M* | *Md* | *SD* | *M* | *Md* | *SD* | *M* | *Md* | *SD* | *M* | *Md* | *SD* | *M* | | *Md* | *SD* | *M* | | *Md* | *SD* |  |
| Warmth | 5.12 | 5.33 | 1.03 | 6.01 | 6.17 | 0.84 | 5.37 | 5.50 | 0.91 | 6.24 | 6.33 | 0.65 | 4.54 | | 4.50 | 1.07 | 5.45 | | 5.50 | 0.99 |  |
| Morality | 5.10 | 5.33 | 1.08 | 5.93 | 6.00 | 0.91 | 5.34 | 5.67 | 0.96 | 6.15 | 6.00 | 0.75 | 4.54 | | 4.67 | 1.14 | 5.37 | | 5.33 | 1.04 |  |
| Sociability | 5.14 | 5.33 | 1.13 | 6.09 | 6.33 | 0.89 | 5.40 | 5.67 | 1.00 | 6.32 | 6.33 | 0.69 | 4.53 | | 4.33 | 1.20 | 5.52 | | 5.67 | 1.08 |  |
| Competence | 5.26 | 5.33 | 1.07 | 5.85 | 6.00 | 0.92 | 5.49 | 5.67 | 0.92 | 6.06 | 6.00 | 0.78 | 4.71 | | 4.67 | 1.18 | 5.34 | | 5.33 | 1.03 |  |
| Similarity | 4.31 | 4.00 | 1.28 | 5.01 | 5.00 | 1.28 | 4.61 | 5.00 | 1.12 | 5.31 | 5.00 | 1.12 | 3.59 | | 4.00 | 1.33 | 4.26 | | 4.00 | 1.35 |  |
| Attractiveness | 4.52 | 5.00 | 1.37 | 4.90 | 5.00 | 1.27 | 4.83 | 5.00 | 1.22 | 5.14 | 5.00 | 1.12 | 3.79 | | 4.00 | 1.43 | 4.30 | | 4.00 | 1.40 |  |
| Rapport |  | | | 4.32 | 4.00 | 0.77 |  | | | 4.50 | 5.00 | 0.62 |  | | | | 3.87 | | 4.00 | 0.91 |  |
| In syncness |  | | | 3.97 | 4.00 | 0.91 |  | | | 4.19 | 4.00 | 0.74 |  | | | | 3.42 | | 4.00 | 1.04 |  |

*Descriptive Statistics of Evaluations during the interactive stage per Partner Selection categories*

# **Supplementary Note 6: Distribution of Person Perceptions, Partner Selection and distributional change between stages**

**Fig 5.***
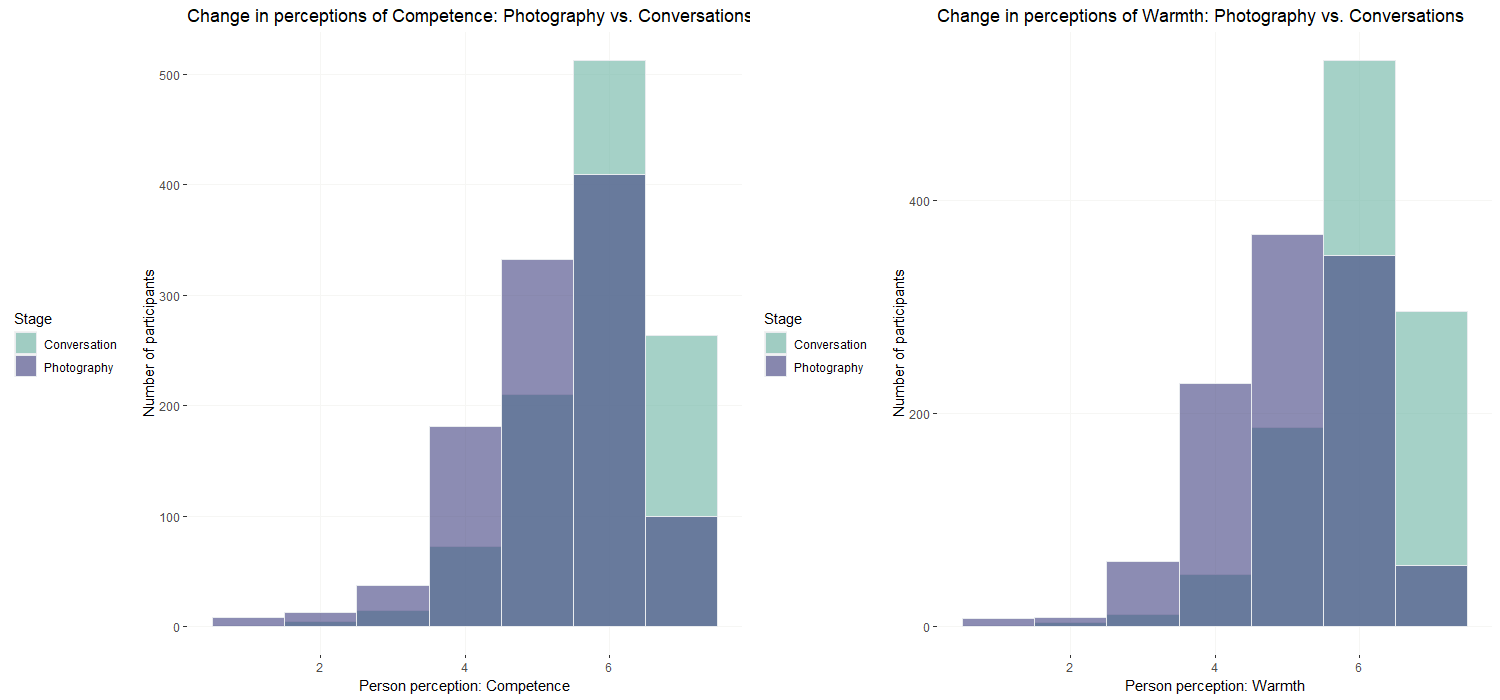
* *Distribution of Person Perceptions of Warmth and Competence between two stages: Photographs and Conversation*

**Fig 6.***
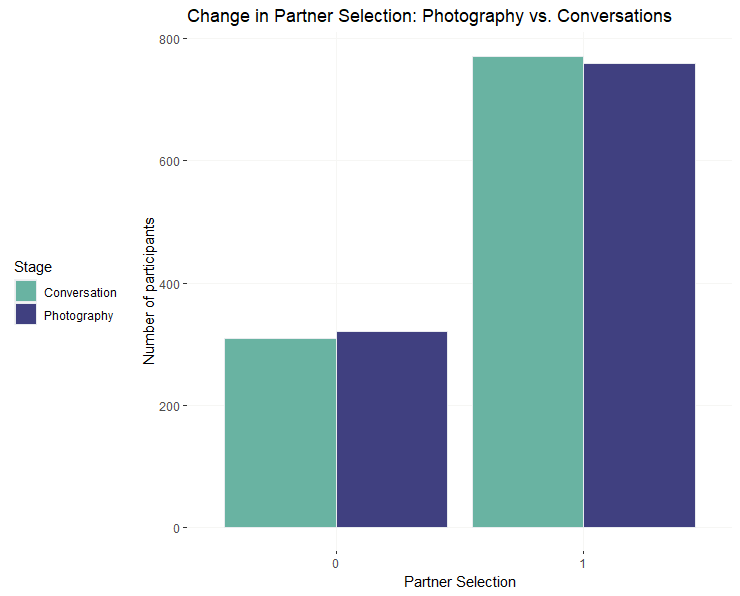
* *Changes in Partner Selection between Photographs and Conversations Stages*

# **Supplementary Note 7: Correlation of Intake Session Variables**

**Table 3**

*Correlation Matrix with Spearman correlation coefficients of all variables measured during the Intake Session (N = 276)*

| Category | Variable | 1 | 2 | 3 | 4 | 5 | 6 | 7 | 8 | 9 | 10 | 11 | 12 |
| --- | --- | --- | --- | --- | --- | --- | --- | --- | --- | --- | --- | --- | --- |
| Personality (HEXACO) | 1. Honesty-Humility | 1 |  |  |  |  |  |  |  |  |  |  |  |
|  | 2. Emotionality | .05 | 1 |  |  |  |  |  |  |  |  |  |  |
|  | 3. Agreeableness | .31*** | -.11* | 1 |  |  |  |  |  |  |  |  |  |
|  | 4. Extraversion | .07 | -.26*** | .20** | 1 |  |  |  |  |  |  |  |  |
|  | 5. Conscientiousness | .23*** | -.01 | .05 | .28*** | 1 |  |  |  |  |  |  |  |
|  | 6. Openess | .06 | -.05 | .04 | .24*** | .13* | 1 |  |  |  |  |  |  |
| Social Anxiety | 7. SIAS | -.18** | .20** | -.18** | -.68*** | -.25*** | -.20** | 1 |  |  |  |  |  |
| Trust | 8. Benevolence | .35*** | .21*** | .45*** | .32*** | .22*** | .26*** | -.26*** | 1 |  |  |  |  |
|  | 9. Ability | .01 | -.25*** | .12* | .54*** | .34*** | .24*** | -.37*** | .25*** | 1 |  |  |  |
|  | 10. Integrity | .30*** | -.06 | .19** | .40*** | .43*** | .31*** | -.33*** | .45*** | .41*** | 1 |  |  |
|  | 11. Propensity | .17** | .08 | .29*** | .07 | .11 | .06 | -.10 | .35*** | .15* | .24*** | 1 |  |
| Intelligence | 12. UCMRT | -.03 | .05 | .02 | -.02 | .04 | .09 | .06 | -.01 | -.12 | -.04 | -.02 | 1 |
| Prosocial behavior | 13. SVO | .34*** | .06 | .17** | .06 | 0 | .18** | -.18** | .29 | -.05 | .14* | .15* | .05 |
|  | Note: '***' = p < .001; '**' = p < .01; '*' = p < .05 | | | | | | | | | | | |  |

# **Supplementary Note 8: Situational Affordances, Person Perceptions and Partner Selection**

| CONTROL MODEL |  |  |  |  |  |  |
| --- | --- | --- | --- | --- | --- | --- |
|  | Estimate | *SE* | *z* | *p* | R²marginal | R²conditional |
| Intercept | 2.23 | 0.89 | 2.50 | .013 | .271 | .501 |
| Batch Size | -0.21 | 0.16 | -1.34 | .181 |  |  |
| Round ID | -0.02 | 0.04 | -0.44 | .660 |  |  |
| Session: Photograph | 0.62 | 0.12 | 4.93 | < .001 |  |  |
| Similarity | 0.97 | 0.09 | 11.34 | < .001 |  |  |
| Attractiveness | 0.60 | 0.08 | 7.38 | < .001 |  |  |
| Number of observation = 2160, otherPID: 279, subjectID = 279 | | | | | | |

**Table 4**

*Results from the Logistic Mixed Models Regression control model predicting Partner Selection*

***Table 5***

|  | Estimate | *SE* | *z* | *p* | R²marginal | R²conditional | *∆R²* |
| --- | --- | --- | --- | --- | --- | --- | --- |
| Intercept | 1.29 | 1.00 | 1.30 | .195 | .368 | .597 | .097 |
| Batch Size | -0.11 | 0.17 | -0.62 | .533 |  |  |  |
| Round ID | -0.00 | 0.04 | -0.01 | .989 |  |  |  |
| Session: Photograph | 1.28 | 0.16 | 8.22 | < .001 |  |  |  |
| Similarity | 0.63 | 0.10 | 6.43 | < .001 |  |  |  |
| Attractiveness | 0.39 | 0.09 | 4.45 | < .001 |  |  |  |
| Competence | 1.00 | 0.15 | 6.69 | < .001 |  |  |  |
| Warmth | 0.57 | 0.15 | 3.75 | < .001 |  |  |  |
| Task type: Warmth | 0.13 | 0.22 | 0.58 | .561 |  |  |  |
| Competence*Task Type: Warmth | -1.20 | 0.20 | -6.04 | < .001 |  |  |  |
| Warmth*Task Type: Warmth | 0.38 | 0.19 | 2.07 | .038 |  |  |  |
| Number of observation = 2160, otherPID: 279, subjectID = 279 | | | | |  |  |  |

*Results of the hypothesized Logistic Mixed Model predicting Partner Selection*

# **Supplementary Note 9: Results from the Model using two different facets of Warmth: Sociability and Morality**

**Table 7**

*Results of the Logistic Mixed Model predicting Partner Selection when using two facets of Warmth: Sociability and Morality*

|  | Estimate | *SE* | *z* | *p* | R²marginal | R²conditional |
| --- | --- | --- | --- | --- | --- | --- |
| Intercept | 1.34 | 1.01 | 1.33 | .184 | .368 | .600 |
| Round ID | 0.00 | 0.04 | 0.02 | .981 |  |  |
| Session: Photograph | 1.29 | 0.16 | 8.26 | < .001 |  |  |
| Similarity | 0.64 | 0.10 | 6.49 | < .001 |  |  |
| Attractiveness | 0.38 | 0.09 | 4.20 | < .001 |  |  |
| Batch Size | -0.11 | 0.18 | -0.65 | .513 |  |  |
| Competence | 1.09 | 0.16 | 6.99 | < .001 |  |  |
| Sociability | 0.62 | 0.16 | 3.91 | < .001 |  |  |
| Morality | -0.12 | 0.20 | -0.62 | .536 |  |  |
| Task type: Trust Task | 0.11 | 0.22 | 0.53 | .600 |  |  |
| Competence*Task Type: Trust Task | -1.30 | 0.21 | -6.32 | < .001 |  |  |
| Sociability*Task Type: Trust Task | -0.14 | 0.21 | -0.68 | .498 |  |  |
| Morality*Task Type: Trust Task | 0.66 | 0.25 | 2.67 | .008 |  |  |
| Number of observation = 2160, otherPID: 279, subjectID = 279 | | | | |  |  |

# **Supplementary Note 10: Partner Selection for Cooperation or Exploitation**

**Table 9**

*Results of the Control and Hypothesized Robust Linear Mixed Model predicting Cooperative Behavior*

| 1. CONTROL MODEL | | | | | | | |
| --- | --- | --- | --- | --- | --- | --- | --- |
|  | *b* | *SE* | *t* | *p* |  | R²marginal | R²conditional |
| Intercept | 10.90 | 2.72 | 4.01 | < .001 |  | .017 | .855 |
| Round | 0.06 | 0.04 | 1.45 | .149 |  |  |  |
| Batch Size | -0.82 | 0.50 | -1.65 | .100 |  |  |  |
| Number of observation: 536, GlobalPID: 138 | | | | |  |  |  |
| 2. HYPOTHESIZED MODEL | | | | | | | |
|  | *b* | *SE* | *t* | *p* | ∆R² | R²marginal | R²conditional |
| Intercept | 8.05 | 2.58 | 3.12 | .002 | .074 | .091 | .830 |
| Round | 0.05 | 0.05 | 1.04 | .301 |  |  |  |
| Batch Size | -0.59 | 0.47 | -1.25 | .213 |  |  |  |
| Partner Selection: Selected | 2.20 | 0.17 | 12.99 | < .001 |  |  |  |
| Number of observation: 536, GlobalPID: 138 | | | | |  |  |  |

**Table 10**

*Results of the Linear Mixed model predicting Other’s Cooperative Behavior*

|  | *b* | *SE* | *t* | *p* | R²marginal | R²conditional |
| --- | --- | --- | --- | --- | --- | --- |
| Intercept | 6.19 | 0.32 | 19.24 | < .001 | .022 | .852 |
| Round | 0.09 | 0.06 | 1.45 | .198 |  |  |
| Batch Size | -0.44 | 0.28 | -1.56 | .120 |  |  |
| Warmth | 0.15 | 0.10 | 1.51 | .132 |  |  |
| Partner Selection:Selected | 0.36 | 0.18 | 2.01 | .045 |  |  |
| Number of observation: 536; otherPID: 138 | | | | |  |  |

# **Supplementary Note 11: (Mis-)Alignment in self- and other- person perceptions and partner selection**

To investigate whether there is an alignment between how individuals see themselves and how they are evaluated by others, we assess whether self-reported traits like personality, social anxiety, prosocial behavior, benevolence, ability, and intelligence were aligned with others' perceptions of warmth or competence. Two linear mixed models were fitted for predicting others’ perceptions of warmth and competence while accounting for subject and person effects as random intercepts.

We did not observe a strong alignment between self-reported personality traits and their interaction partner’s perceptions of warmth. In case of predicting warmth perceptions, we did not find any alignment with previously hypothesized variables, including Extraversion (*b* = 0.05, *SE* = 0.03, *t* = 1.79, *p* = .075), Agreeableness (*b* = -0.00, *SE* = 0.03, *t* = -0.02, *p* =.986), Honesty-Humility (*b* = 0.00, *SE* = 0.02, *t* = 0.20, *p* = .840), Social Value Orientation (*b* = -0.01, *SE* = 0.02, *t* = -0.51, *p* = .609), Psychopathy (*b* = -0.04, *SE* = 0.03, *t* = -1.33, *p* = .185), Social Anxiety (*b* = 0.01, *SE* = 0.03, *t* = 0.37, *p* = .710) and Benevolence (*b* = 0.02, *SE* = 0.03, *t* = 0.65, *p* = .516). However, we did observe that objective UCMRT scores were significant predictors of perceptions of competence. Specifically, intelligence scores were positively associated with others’ perceptions of their competence (*b* = 0.06, *SE* = 0.02, *t* = 2.76, *p* = .006). Furthermore, perceptions of one’s ability were not associated with other’s perceptions of competence (*b* = 0.01, *SE* = 0.03, *t*(184) = 0.29, *p* = .770).

**Table 12**

*Results of the Linear Mixed Models predicting Person Perception of Warmth*

| 1. Control Model | | | | | | |
| --- | --- | --- | --- | --- | --- | --- |
|  | *Estimate* | *SE* | *t* | *p* | R²marginal | R²conditional |
| Intercept | 6.06 | 0.04 | 152.43 | < .001 | .211 | .553 |
| Session: Photograph | -0.86 | 0.03 | -31.01 | < .001 |  |  |
| Batch Size | -0.04 | 0.03 | -1.03 | .303 |  |  |
|  | | | | | | |
|  | *Estimate* | *SE* | *t* | *p* | R²marginal | R²conditional |
| Intercept | 6.06 | 0.04 | 152.76 | < .001 | .218 | .555 |
| Session: Photograph | -0.87 | 0.03 | -31.03 | < .001 |  |  |
| Batch Size | -0.04 | 0.03 | -1.07 | .285 |  |  |
| Extraversion | 0.05 | 0.03 | 1.79 | .075 |  |  |
| Agreeableness | -0.00 | 0.02 | -0.02 | .986 |  |  |
| Honesty-Humility | 0.00 | 0.02 | 0.20 | .840 |  |  |
| Social Value Orientation | -0.01 | 0.02 | -0.51 | .609 |  |  |
| Psychopathy | -0.04 | 0.03 | -1.33 | .185 |  |  |
| Social Anxiety | 0.01 | 0.03 | 0.37 | .710 |  |  |
| Benevolence | 0.02 | 0.03 | 0.65 | .516 |  |  |
| Number of observation = 2160, otherPID: 279, subjectID = 279 | | | | |  |  |

**Table 14**

*Results of the Robust Linear Mixed Model predicting Person Perception of Competence*

| 1. Control Model |  |  |  |  |  |  |
| --- | --- | --- | --- | --- | --- | --- |
|  | *Estimate* | *SE* | *t* | *p* | R²marginal | R²conditional |
| Intercept | 5.91 | 0.04 | 136.69 | < .001 | 0.092 | 0.539 |
| Session: Photograph | -0.55 | 0.03 | -19.92 | < .001 |  |  |
| Batch Size | -0.05 | 0.04 | -1.41 | < .001 |  |  |
|  | *Estimate* | *SE* | *t* | *p* | R²marginal | R²conditional |
| Intercept | 5.93 | 0.05 | 139.67 | < .001 | 0.104 | 0.534 |
| Session: Photograph | -0.58 | 0.03 | -18.16 | < .001 |  |  |
| Batch Size | -0.06 | 0.04 | -1.46 | .145 |  |  |
| UCMRT | 0.06 | 0.02 | 2.76 | .006 |  |  |
| Ability | 0.04 | 0.02 | 1.66 | .098 |  |  |
| Number of observation: 1714; OtherPID: 271; GlobalPID: 224 | | | |  |  |  |

# **Supplementary Note 12: MiniRocket**

MiniROCKET applies many convolutional kernels to capture local motifs and patterns within the time-series data. Here, each kernel corresponds to a specific filter, which is slid across the time series. At each position, the dot product between the kernel and the corresponding part of the time-series is computed. Such values are then aggregated over each feature map of a specific kernel, called pooling. For the MiniROCKET multivariate model we used fixed values for the hyperparameters. Firstly, the number of feature representations was produced by 10000 kernels, while the maximum dilation per kernel was set to the default value of 32, as recommended (Dempster et al., 2021). The input to the model was a highly dimensional matrix where each video-audio recording of each participant (n videos = 1022) was represented as a high dimensional matrix of 5400 rows (timepoints) x 754 columns (OpenSmile and OpenFace features) (see Figure 2). All videos were concatenated to form one matrix (5518800x754) which was fed into a MiniROCKET multivariate, producing a reduced matrix where each video-audio recording (1022 rows) was associated with a row vector of 9996 containing extracted MiniROCKET time-series features (1022x9996). These time-series features were further used to a machine-learning algorithm.

# **Supplementary Note 13: Nested Cross-validation**

Cross-validation techniques are used in machine learning to estimate the performance of the model and select the best hyperparameters (i.e., the best model). It has two loops, thus the name nested. In the outer loop, the original dataset is divided into multiple subsets (k = number of sets), typically using k-fold cross-validation. Usually, the number of subsets is defined by the researchers. In each iteration of the outer loop, one subset is held out as the test set, and the model is trained on the remaining training data. The performance of the model is evaluated on the held-out test set, yielding an estimate of its generalization performance. Thus, the outer loop is used for model evaluation. Within each iteration of the outer loop, an inner loop is used for hyperparameter tuning. The training subset generated in the outer loop is further divided into smaller subsets, generating a development subset and a reduced test set, again using k-fold cross-validation. The number of splits (k) used in the inner loop doesn’t have to be the same as in the outer loop. Then, different development subsets are used to evaluate different hyperparameter combinations defined by the hyperparameter grid. The best model configuration is selected for each development subset. Then, the best hyperparameter configuration that performs well on average is selected and used on the outer loop, where it is evaluated on the held-out test set. However, before being evaluated on the outer loop test sets, the best model is re-trained on the entire training set generated in the outer loop and then evaluated on the test set. This process is repeated for each iteration of the outer loop resulting in multiple evaluations of the model’s performance (James, Witten, Hastie & Tibshirani, 2023).

# **Supplementary Note 14: EULA**

**Scope**

This End User License Agreement (hereinafter: "License") is granted by the {anonymous} (hereinafter: "Licensor") to the End User. This License is an Open-Source License.

**Material under License**

The materials under License (hereinafter: "Dataset") is a dataset composed of images, audio-video recordings, and surveys recorded via an online survey and online conferencing study. The dataset contains raw and processed data as well as any derived work, products, or services based on all or part of the data. All data contained within the Dataset have been collected and processed in accordance with the laws applicable in {anonymous}.

**Copyright**

The Dataset is the sole property of the Licensor and is protected by copyright. The Licensor reserves all rights to the use and distribution of the Dataset. The Dataset shall remain the exclusive property of the Licensor. The End User acquires no ownership, rights, or title of any kind with regard to the Dataset.

**License**

Following the signature of this License, the Dataset is freely available to the benefit of the End User. The Licensor grants the End user the right to use the Dataset, for academic non-governmental research with non-commercial purposes only.

The End User shall be responsible for any infringement of the present License by one of their subsidiaries and/or student's. The End User may only disclose, give access, and/or transfer the rights related to the Dataset to subsidiaries and/or students under the following conditions:

- A copy of the present License has already been transferred to them;
- The subsidiaries/students have fully read and understood all terms and conditions of the present License;
- Access to the Dataset is granted under the close supervision of the End User;
- The access to the Dataset is granted under the sole responsibility of the End User.

The Licensor grants to the End User the temporary rights to reproduce, adapt, arrange and modify by any means the Dataset. The Licensor grants to the End User the right to rework and build upon the original Dataset, or any component thereof, as necessary or desirable for research or technology development activity and create derivative products or services for the End User's internal research and development. The End User is permitted to make a copy of the Dataset for these purposes. Any such copy has to be deleted after active research ceases or the End User switches institutions, after sending it to the Licensor. This License is deemed non-exclusive and non-transferable to third parties.

**Access**

The End User may only use the Dataset after this License has been signed and returned to the Licensor. The End User must return the signed and dated License by email, in PDF format to the Licensor at the following address {email address} under the subject "Requesting access to the dataset".

The End User will receive access to semi-anonymized data and will not attempt to establish the identity, nor attempt to make direct contact with subjects or staff at sites concerning the specific results of individual subjects, nor attempt to contact any of the subjects in general.

**Copyleft and Open Science**

The Licensor grants to the End User the License defined under section 2 under the sole condition that any derivative work, product, services, scientific developments of any kind, or any improvement or modification on all or part of the dataset shall be made freely available to other End Users. A copy of any modification of the Dataset shall be submitted to the Licensor at the following address: {email address} The End User shall ensure that, in the event of the modification of the Dataset, the substance of the Material is not altered, the facility still operates and performs its part in an intact meaningful way. Any violation of this clause will give rise to immediate legal prosecution.

**Distribution**

The End User shall not, without any authorization of the Licensor, permanently or temporarily, transfer, distribute, or broadcast all or part of the Dataset to third parties. The End User will need to ask and send all request for the distribution to the Licensor at the following address: {email address}

**Research**

Research includes all types of scientific research involving in detailed study of a subject to discover new information or understand the subject better. Here, we also emphasize the need for ethical research that applies the fundamental ethical principles to scientific research, including research design, implementation of research, the use of resources, and research outputs.

**Commercial use**

Any commercial use of the Dataset is strictly prohibited. Commercial use of the Dataset includes, but is not limited to:

- Developing commercial systems
- Providing the efficiency of commercial systems
- Testing commercial systems
- Using screenshots of subjects from the Dataset in the advertisement
- Selling data or making any commercial use of the dataset
- Broadcasting data from the Dataset

Any violation of this clause will give rise to immediate legal prosecution by the Licensor. Any damages and/or unfair enrichment of the End User due to the breach of the License shall be immediately restituted to the Licensor together with the derivative works, products, and services based on all or part of the Dataset.

**Publications**

The End User shall reference the Dataset in publications. Publications include, but are not limited to:

- Research papers

- Reports in any form (i.e. posters)

- Articles

- Presentation for educational and conference purposes

All uses of images and videos from the Dataset for demonstration/visualization purposes (articles, presentations, posters) must ensure the privacy of the participants in the Dataset, such as covering their faces or blurring out the identifiable part of the scenery.

All publications that report on research that use the Dataset will acknowledge this as follows: Portions of the research in this paper used the Paco Dataset made available by the University of anonymous.

**Illegal or criminal use of the dataset**

Any illegal or criminal use of the Dataset by the End User is strictly prohibited.

**Legal Disclaimer**

The Dataset is granted without any warranty. Licensor shall not be held responsible for any damage (physical, financial or otherwise) caused by the use of the Dataset. Licensor shall not be held responsible for any illegal or criminal use of the Dataset by the End User.

**Jurisdiction**

The License is subject to and interpreted in accordance with {anonymous} Law. Any claim arising on the basis of this License shall exclusively be submitted to the Courts of {anonymous}

**Amendments**

The Licensor is allowed to amend this License at any time without prior consent of the End User. The End User shall be informed about the changes and given the option to opt out of this License within 10 days to the Licensor at the following address: {email address} If the End User does not notice the Licensor within 10 days and if the provided amendment is not substantial, the amendment to the License will be fully applicable to the End User.

**Warranties**

The End User warrants that they are authorized signatory, adult and not legally forbidden to enter into this License.

By signing this agreement, the End User warrants that they have read and understood all elements contained in the previous sections. Specifically, by signing this document, the End User acknowledges and agrees to the following conditions:

- The Dataset includes personal data with privacy protection.
- The End User is responsible for the correct (i.e. as described in this document) use of the Dataset.
- The Dataset may not be further distributed.
- The Dataset may only be used for research purposes.
- The Dataset may not be used with the intention of identifying subjects included in the Dataset
- The End User will be taken the rights of using the Dataset in case of misuse.
- The End User should take sufficient security measures for protecting the Dataset, with strong emphasis on personal data.

By signing the License, the End User Engages to strictly respect the conditions disclosed in this document and to respect all the laws applicable in {anonymous} in relation to (personal) data protection.

# **References**

Berg J, Dickhaut J, McCabe K. Trust, reciprocity, and social history. Games and economic behavior. 1995 Jul 1;10(1):122-42.

Eyben F, Wöllmer M, Schuller B. Opensmile: the munich versatile and fast open-source audio feature extractor. InProceedings of the 18th ACM international conference on Multimedia 2010 Oct 25 (pp. 1459-1462).

James G, Witten D, Hastie T, Tibshirani R, Taylor J. An introduction to statistical learning: With applications in python. Springer Nature; 2023 Jun 30.
