# Supplementary material for: Partner perceptions during brief online interactions shape partner selection and cooperation
Source: PLoS One. 2025 Apr 9;20(4):e0318137. doi: 10.1371/journal.pone.0318137 (PMC11981216; doi:10.1371/journal.pone.0318137)
Supplement: Attachment — Submitted filename: Response to Reviewers.docx [file pone.0318137.s003.docx]

Dear Dr. Daniel Redhead,

Please find enclosed the manuscript entitled “*Partner Perceptions During Brief Online Interactions Shape Partner Selection and Cooperation*”, that we have revised for further consideration at *PLOS ONE*. We greatly appreciate yours and the reviewers’ constructive comments on our manuscript. We used these comments to further strengthen the paper. Below we detail how we have changed the manuscript to address each of the comments.

Below we detail our responses to the editor and reviewers’ comments. We include the comments in italics and our response in normal font. When we have made changes to the manuscript, we either insert the change in the cover letter or provide the page number where the change can be found in the paper. We hope you find this information easy to navigate.

To our knowledge, we have followed *PLOS ONE* guidelines for manuscript submissions. Each author has read the manuscript, agrees with the Journal’s deposition requirements, and have no conflict of interests.

Thank you for further considering our paper, and we look forward to hearing from you.

Sincerely,

Tiffany Matej Hrkalovic

*Editor’s Comments*

*Thank you for submitting your manuscript to PLOS ONE. After careful consideration, we feel that it has merit but does not fully meet PLOS ONE’s publication criteria as it currently stands. Therefore, we invite you to submit a revised version of the manuscript that addresses the points raised during the review process.

I apologize for the delayed review process for your manuscript, it was tough to secure reviewers. First, I’d like to commend the authors on the manuscript. I read the manuscript in detail, and found it well-written, answering an important question that is highly relevant to several disciplines in a careful and considered manner. The experimental paradigm is relatively novel, and the treatment and analysis of these quite complex data is appropriate and generates new insights into peer perceptions and partner choice. My enthusiasm is echoed by both reviewers, who recommend minor revisions and several clarifications. Reviewer 1 provides detailed comments and suggestions that are important to address during revision. Reviewer 2 notes how impressive the manuscript is and, while they correctly note that there is a lot in the manuscript (and suggest potentially splitting it into two separate manuscripts), I believe that some careful addition to the discussion of the results could ensure that the focus is coherent. Please do, however, respond to all of Reviewer 2's points in your revision. Additionally, I provide some feedback below from my reading that I think will strengthen the argumentation and clarity presented throughout the manuscript. 

Please include the following items when submitting your revised manuscript:*

- *A rebuttal letter that responds to each point raised by the academic editor and reviewer(s). You should upload this letter as a separate file labeled 'Response to Reviewers'.*
- *A marked-up copy of your manuscript that highlights changes made to the original version. You should upload this as a separate file labeled 'Revised Manuscript with Track Changes'.*
- *An unmarked version of your revised paper without tracked changes. You should upload this as a separate file labeled 'Manuscript'.*

*If you would like to make changes to your financial disclosure, please include your updated statement in your cover letter. Guidelines for resubmitting your figure files are available below the reviewer comments at the end of this letter.

We look forward to receiving your revised manuscript.*

*Kind regards,*

*Daniel Redhead
Academic Editor
PLOS ONE*

Journal requirements:

When submitting your revision, we need you to address these additional requirements.

1. Please ensure that your manuscript meets PLOS ONE's style requirements, including those for file naming. The PLOS ONE style templates can be found at

https://journals.plos.org/plosone/s/file?id=wjVg/PLOSOne_formatting_sample_main_body.pdf and

<https://journals.plos.org/plosone/s/file?id=ba62/PLOSOne_formatting_sample_title_authors_affiliations.pdf>

Authors reply on J1: We formatted the main body and the author affiliations as indicated in the files.

2. Please include a caption for figure 3.

Authors reply on J2: Now, all figures including figure 3 have their corresponding captions, see p.25.

**Examples: “*Fig 3. Interaction Between the Type of Task and Person Perceptions of Warmth and Competence Predicting Partner Selection****.* *The figure illustrates how the type of task moderates the relationship between perceptions of warmth and competence in predicting partner selection. Specifically, participants perceived as higher in competence were more likely to be selected as partners for the* ***Joint Competence Task****, but this relationship was not observed for the* ***Joint Trust Task****. Participants perceived as higher in warmth were more likely to be selected as partners for both tasks. However, partner selection was slightly stronger for the* ***Joint Trust Task*** *compared to the* ***Joint Competence Task*.”**

3. We note that the grant information you provided in the ‘Funding Information’ and ‘Financial Disclosure’ sections does not match.

When you resubmit, please ensure that you provide the correct grant numbers for the awards you received for your study in the ‘Funding Information’ section.

4. Thank you for stating the following financial disclosure:

 [D.P (# 864519) awarded from the ERC Consolidator grant (https://erc.europa.eu/apply-grant/consolidator-grant)

T.M.H. (funded via Hybrid intelligence Center funded by the Dutch Ministry of Education) grant number 024.004.022 (https://www.nwo.nl/en)].

Please state what role the funders took in the study.  If the funders had no role, please state: ""The funders had no role in study design, data collection and analysis, decision to publish, or preparation of the manuscript.""

If this statement is not correct you must amend it as needed.

Please include this amended Role of Funder statement in your cover letter; we will change the online submission form on your behalf.

Authors reply on J3 and J4: We corrected and stated the role of the grant recipients.

5. We note that you have indicated that there are restrictions to data sharing for this study. PLOS only allows data to be available upon request if there are legal or ethical restrictions on sharing data publicly. For more information on unacceptable data access restrictions, please see http://journals.plos.org/plosone/s/data-availability#loc-unacceptable-data-access-restrictions.

Before we proceed with your manuscript, please address the following prompts:

a) If there are ethical or legal restrictions on sharing a de-identified data set, please explain them in detail (e.g., data contain potentially identifying or sensitive patient information, data are owned by a third-party organization, etc.) and who has imposed them (e.g., a Research Ethics Committee or Institutional Review Board, etc.). Please also provide contact information for a data access committee, ethics committee, or other institutional body to which data requests may be sent.

b) If there are no restrictions, please upload the minimal anonymized data set necessary to replicate your study findings to a stable, public repository and provide us with the relevant URLs, DOIs, or accession numbers. For a list of recommended repositories, please see

https://journals.plos.org/plosone/s/recommended-repositories. You also have the option of uploading the data as Supporting Information files, but we would recommend depositing data directly to a data repository if possible.

We will update your Data Availability statement on your behalf to reflect the information you provide.

Authors reply to J5: We will upload the self-report part of the dataset on OSF. However, the data that was used for training machine learning models is too big in size to share on OSF (e.g., data from OpenSmile and OpenFace). Thus, we can share it with individuals after they sign the EULA. Also, due to the high sensitivity of the raw audio-and video-data cannot be shared publicly, only upon request from an accredited academic institution and signing the EULA.

6. We are unable to open your Supporting Information file [Fig1.eps, Fig2.eps, Fig3.eps and Fig4.eps]. Please kindly revise as necessary and re-upload.

Authors reply on J6: Now, we share all the figures in .tiff format with appropriate dpi’s. However, just a note, these are figures that need to be presented in the main manuscript.

7. Please include captions for your Supporting Information files at the end of your manuscript, and update any in-text citations to match accordingly. Please see our Supporting Information guidelines for more information: http://journals.plos.org/plosone/s/supporting-information.

Authors reply on J7: As a supporting file, we only have the supplementary materials, which we re-named into S1 Files, as such these files shouldn’t require any captioning, as guided by previous PLOS ONE submissions that had S1 Files as .doc files.

8. Please review your reference list to ensure that it is complete and correct. If you have cited papers that have been retracted, please include the rationale for doing so in the manuscript text, or remove these references and replace them with relevant current references. Any changes to the reference list should be mentioned in the rebuttal letter that accompanies your revised manuscript. If you need to cite a retracted article, indicate the article’s retracted status in the References list and also include a citation and full reference for the retraction notice.

Authors reply on J8: We reviewed our reference list, and to our knowledge, the list doesn’t contain any retracted papers. We also checked if the list of references is complete.

Additional Editor Comments:

P.3, line 59 onwards: While warmth-competence is a leading theory in social psychology, there are many other (evolutionary) theories on partner choice. Many of these theories of (reputation-based) partner-choice are based on ideas of an individual’s ability and willingness to confer benefits to others but don’t use the terminology “warmth” and “competence” (e.g., Baumard et. al. 2013; Fu et. al., 2008; Roberts 2015). I think that it is important to disentangle ideas of an individual’s ability and willingness to confer benefits from the warmth-competence framework. This could simply be done by having one sentence that outlines that theory points to partner choice being guided by perceptions of an individual’s ability and willingness to cooperate with others (acknowledging the diversity of relevant frameworks), and then follow this with the remainder of the section about warmth-competence being one of such frameworks.

Authors reply to E1a: Thank you for highlighting this point. We now take the approach you suggest in the text, as well as referencing previous work on modeling partner selection. Specifically, we integrate two different schools of thought on the role of perceptions in partner selection and social behavior. However, we focus on the social evaluation theories, as it is a useful lens that applies to social evaluations in everyday life. Additionally, our measurement tools for perceptions of warmth and competence are taken from studies that are based on Social evaluation models. Nevertheless, we integrate both perspectives in the paper.

For example, we rewrote the second part of the introduction to acknowledge both perspectives. See p.3-4, lines: 68-77: *“When selecting partners, two key evaluation dimensions are important: the likelihood and ability of potential partners to act cooperatively or selfishly [4, 6, 9]. Prior research empirically highlighted the importance of proxies of these dimensions in partner selection decisions [10], especially in cooperative tasks that involve conflicts between individual and collective interests, known as social dilemmas [11-12]. Social evaluation models also emphasize these dimensions as the foundation for how people assess others. They are commonly referred to as warmth (willingness to cooperate) and competence (ability to contribute effectively) [13]. In everyday interactions, these perceptions are shaped by various social cues, such as non-verbal behaviors like smiling or voice pitch, which consistently enhance perceptions of warmth and competence in interactions with strangers [14-17].”*

The focus on non-verbal behaviors is necessary, but misses an existing literature on verbal cues (e.g., vocal synchrony: Bowling et. al. 2013; vocal frequencies: Knowles & Little, 2016). This should be acknowledged. Alongside this, there are several examples of how (social) behaviors related to cooperation inform perceptions of an individual’s ability and willingness to confer benefits (e.g., von Rueden et. al., 2019; Redhead et al., 2021)—and partner choice more generally to others in real-world settings (e.g., Redhead, Dalla Ragione & Ross, 2023, Redhead et al., 2024). Many of such studies are not framed around ideas of warmth-competence, but are certainly relevant to the discussion of theory and previous empirical work throughout the introduction.

Authors reply to E1b: Thank you for sharing additional literature. Regarding the literature on non-verbal behaviors, we integrated the reference for Knowles & Little, 2016. We did not include literature in this paper on synchrony and mimicry for two reasons. Firstly, in this paper, we are focused on the “what” of non-verbal behaviors. The dynamic of these non-verbal behaviors (i.e., “how”), such as synchrony and mimicry, will be addressed as a separate aspect of the conversation and will be investigated in other papers.

Secondly, we acknowledge that there are many other social cues and behaviors that people use to inform their evaluations and cooperation, but then we note that we specifically focus on non-verbal behavior and their role in informing perceptions of warmth and competence. To address this comment, we make this statement clearer in the current paper and acknowledge some of the literature that we mentioned in the comment (e.g., von Rueden et al., 2019, Redhead, D. et al., 2023). Our goal was to incorporate the perspectives that are present in both social evaluation and evolutionary models but keep an emphasis on the social evaluation models.

*For example, p.5. lines 98-104, we state: “A key mechanism of partner selection lies in the ability to evaluate others, form perceptions, and use these perceptions to guide partner choice. These perceptions are shaped by a myriad of social cues [21-22], ranging from reputational information [4] to the partner’s sex and age [24], social status [25, 10] and many more. However, this paper focuses on non-verbal behavior cues [14, 26]. These behavioral cues enable individuals to make quick, though sometimes imperfect, evaluations of others [16, 27]. Nevertheless, these evaluations help individuals navigate social interactions by reducing uncertainty about unknown individuals [22].”*

E2: Please also expand the discussion of limitations related to studies using only online convenience samples, as per Crump et. al. (2013) and Chandler & Shapiro (2016).

Authors reply to E2: Thank you for highlighting this point. We extended our list of limitations and included the limitations that are stated in Crump et al., (2013).

p.33, lines 750 – 755: *“A limitation of this study is the lack of control over participants' recording environments, such as webcams, microphones, and backgrounds—an inherent challenge in online data collection [71]. Efforts were made to mitigate other online study drawbacks. For instance, participant progress was tracked in real time to minimize distractions. While we ensured participants were fully engaged during online interactions, we cannot guarantee their undivided attention while completing questionnaires, which remains a potential limitation [71].”*

Methodology:

E3: Please report the range in age.

Authors reply to E3: We included the age range.

*p.10, line 233-235: “The final number of participants decreased to 279 participants (Female = 154; Male = 122; Non-binary = 2; Unknown = 1; M_age_ = 36.69, SD_age_ = 11.03; Range_age_ = 19 – 67 years old) after excluding participants who dropped out or had incomplete self-reported data.”*

E4: P. 12, lines 253-254: How was the threshold of 2 minutes of conversation decided upon? Was there any systematic patterning of conversations (e.g., were shorter conversations structured by any demographic factors or by combination of the self-report personality traits, and does removing these such conversations significantly change results)? Please elaborate and provide some justification.

Authors reply to E4: Now, we now elaborate how the threshold of 2 minutes of conversation was decided upon. The 2 minutes were taken as the threshold because the participants could end the call (the” hang up” button appeared) after two minutes had passed in in the conversation. Thus, some participants could have the entire conversation in 2 minutes. To acknowledge this, and not penalize them we decided to include all the conversations from 2 minutes and above (maximum duration was 3 minutes) in our analyses. Additionally, there was a maximum duration set of 2 minutes, but sometimes, due to technical issues, conversations did not stop as intended. Thus, we filtered these out.

*p.11, lines 240-243: “The threshold of two minutes was used, as individuals could stop the conversation at two minutes by hanging up the call. Thus, all the conversations that lasted less than two minutes finished earlier due to some technical complications and were not taken into consideration for further analysis.”*

Minor notes

- P. 5, Line 92: I think it should be “partners”.
- P. 7, Line 148: The citation for FeldmanHall & Shenhav (2019) seems to be out of place.
- P. 10, Line 218: This should be past tense (“had a conversation”).
- P. 12, Line 264: I think this should be “after providing written consent”.
- P. 28, Line 589: Typo, please remove “in partner selection”.

Authors reply to Minor Notes: Thank you for the detailed overview of mistakes in the manuscript. We corrected them and made sure they are not repeated in the manuscript.

Suggested References

Baumard, N., André, J. B., & Sperber, D. (2013). A mutualistic approach to morality: The evolution of fairness by partner choice. Behavioral and Brain Sciences, 36(1), 59-78.

Bowling, D. L., Herbst, C. T., & Fitch, W. T. (2013). Social origins of rhythm? Synchrony and temporal regularity in human vocalization. PLoS One, 8(11), e80402.

Chandler, J., & Shapiro, D. (2016). Conducting clinical research using crowdsourced convenience samples. *Annual review of clinical psychology*, *12*(1), 53-81.

Crump, M. J., McDonnell, J. V., & Gureckis, T. M. (2013). Evaluating Amazon's Mechanical Turk as a tool for experimental behavioral research. *PloS one*, *8*(3), e57410.

Fu, F., Hauert, C., Nowak, M. A., & Wang, L. (2008). Reputation-based partner choice promotes cooperation in social networks. Physical Review E—Statistical, Nonlinear, and Soft Matter Physics, 78(2), 026117

Knowles, K. K., & Little, A. C. (2016). Vocal fundamental and formant frequencies affect perceptions of speaker cooperativeness. Quarterly Journal of Experimental Psychology, 69(9), 1657-1675.

Redhead, D., Dhaliwal, N., & Cheng, J. T. (2021). Taking charge and stepping in: Individuals who punish are rewarded with prestige and dominance. Social and Personality Psychology Compass, 15(2), e12581.

Redhead, D., Dalla Ragione, A., & Ross, C. T. (2023). Friendship and partner choice in rural Colombia. Evolution and Human Behavior, 44(5), 430-441.

Redhead, D., Gervais, M., Kajokaite, K., Koster, J., Hurtado Manyoma, A., Hurtado Manyoma, D., ... & Ross, C. T. (2024). Evidence of direct and indirect reciprocity in network-structured economic games. Communications Psychology, 2(1), 44.

Roberts, G. (2015). Partner choice drives the evolution of cooperation via indirect reciprocity. PloS one, 10(6), e0129442.

von Rueden, C. R., Redhead, D., O'Gorman, R., Kaplan, H., & Gurven, M. (2019). The dynamics of men's cooperation and social status in a small-scale society. Proceedings of the Royal Society B, 286(1908), 20191367.

**Reviewer 1:**

1. The potential effects of participant sex and partner sex are not addressed at all. This seems like an obvious oversight for a paper on partner choice and cooperation. In opposite-sex interactions, you might expect partner attractiveness to affect rates of partner selection and cooperativeness in the trust game (e.g. Maestripieri et al., 2017, Behavioral and Brain Sciences). You might also expect participants (especially men) to be more cooperative in the trust game with opposite-sex vs. same sex participants, and there’s evidence that men and women behave differently in same-sex economic games (e.g. men might be engaged in more partner choice/attraction, while women might be more intrasexually competitive; e.g. Eisenbruch et al., 2016, Evolution & Human Behavior; Lucas & Koff, 2013, Evolution and Human Behavior). To the extent possible, it would be interesting to explore the effects of participant sex, whether the trial was same- or opposite-sex, and their interaction. At a minimum, this topic should be discussed as a limitation and/or direction for future research.

Authors reply to R1.1: Thank you for highlighting this aspect, and we completely agree that participants’ sex is another important social cue that could be used as a potential factor in guiding partner selection. However, as you also mentioned there is a lot of work on the effects of sex and dyadic compositionality on behavior and decision-making.

The goals of our paper were to investigate a general effect on perceptions formation, their role in partner selection, and consequently their trade-off due to task affordances, regardless of sex and dyadic compositionally. Thus, to avoid giving these factors only a supplementary acknowledgment, we decide to not do additional analyses to address this topic. However, we thank you for proposing that we should conduct future work on this topic. We now mention the topic in the discussion section of the paper about future work and limitations. That said, if you or the editor think we should more thoroughly address this topic, we can attempt to do that.

See paragraph Future Research:

p.34 lines 768-772: *“Additionally, our study did not account for the sex of the partner and the chooser in making partner evaluations and selection, and we did not consider the dynamics of same-sex versus opposite-sex interactions. Future research could explore these aspects, as opposite-sex interactions and gender differences in partner preferences may influence cooperative behavior [72].”*

2. The fact that people adjust their preferences for warmth and competence to the task type is interesting. It should be connected to the existing literature on relationship-specific social preferences (e.g. Sprecher & Regan 2002, JSPR; Cottrell et al., 2007, JPSP).

Authors reply to R1.2: We are aware of the Cottrell et al., 2007 paper, and indeed it should have also been mentioned in the discussion. Now, we provide a sentence that acknowledges whether the findings were in line with the two papers. Thank you.

p.30. lines 670 – 684: *“Further analysis revealed that in the Joint Trust Task, competence was not a significant predictor of partner selection, whereas warmth was a strong predictor in both tasks, with a higher predictive weight in the Joint Trust Task. These results are in line with prior literature showing the difference in partner selection preferences for cooperative and competitive tasks [21], as well as social preferences in different relationship types [45,67]. For instance, Cottrell et al., (2007) [21] observed that preferences for competence increase in relationship types affording competence, such as study or business partnerships. However, warmth-related traits such as trustworthiness and cooperativeness were preferred in all relationship types, including friends or romantic partners. Findings showing people’s high preferences for warm partners in both tasks are also in line with other existing literature showing the primacy of warmth over competence in social perceptions and partner selection [11-12]. Arguably, morality (i.e., trustworthiness) is the most relevant trait for the Joint Trust Task, and indeed we found that when only doing analyses with the morality items, perceptions of morality were more predictive of partner selection in the Joint Trust Task, relative to the Joint Competence Task.”*

3. At the same time, it’s very interesting the degree to which people did NOT adjust their preferences to the task type. While people’s preference for competence only existed in the joint competence task (very clever task, by the way), the preference for warmth was present in both conditions. In other words, even when the task had no affordance for warmth to affect outcomes at all, people still had a pretty strong preference for warm partners. This is consistent with the existing literature on the primacy of warmth over competence in social perception and partner choice (e.g. Fiske et al., 2007, TiCS; Eisenbruch & Krasnow, 2022; Raihani & Barclay 2016; Robertson, T. E., Krasnow, M., & Lim, J. (2017, May 31–June 3). People prefer those who value them over those who benefit them [Paper presentation]. 29th Annual Human Behavior and Evolution Society Conference, Boise, ID, United States.). The fact that people did this when it was unambiguously irrelevant to the task at hand suggests that people’s partner preferences are partially rationally calibrated to their current situation, partially hardwired (perhaps by evolution).
a. The effect of warmth on partner selection in the joint competence task is misstated on p. 32, lines 682-684. What’s written here contradicts the results reported on p. 25 and Figure 3.

Authors reply to R1.3: Thank you for recognizing the benefit of the joint competence task. We also agree with the thoughts you shared and view of the proposed literature. As a result, we rewrote the paragraph in the discussion where we interpreted these results, and highlighted the literature you mentioned (i.e., Fiske et al., 2007, TiCS; Eisenbruch & Krasnow, 2022; Raihani & Barclay 2016). Additionally, we correct the mistake of the misstated results on p.32. Thank you for noticing this.

For complete revision, see the paragraph that was included in the manuscript under Authors reply to R1.2.

4. It’s interesting that participants’ competence perceptions tracked the partners’ performance in the cognitive task in phase 1 of the study. Did the perceptions of competence track actual performance in the joint competence task? There is a small but growing literature on the ability to accurately detect certain forms of competence from (men’s) faces (e.g. IQ: Kleisner et al., 2014, PLOS ONE; hunting ability: Eisenbruch et al., 2024, EHB; fighting ability: Zilioli et al, 2014, Aggressive Behavior), which is consistent with the accuracy of competence perceptions reported here.

Authors reply to R1.4: It is an interesting result indeed, especially as participants did not know how well they did in the cognitive task in phase 1, so there is no chance of engaging in cheap talk, and purely mentioning their result.

Here we only wanted to see if cooperative behavior aligned with how participants were perceived by others. As the true cooperative measure was the Joint Trust Task, we only correlated the participants’ perceptions of warmth with their actual cooperative behavior. As a result, we did not compute the accuracy in the Joint Competence Task. However, as you mention the accuracy of detecting certain forms of objective competence was also replicated in our paper, as the participants objective performance on the cognitive task was predictive of how these individuals were perceived on competence.

5. The point about the need for social preference studies based on actual interactions (instead of single cues like just faces or voice recordings) is a good one, but there are a small number of existing studies that do that. Eisenbruch et al., 2019 (Adaptive Human Behavior & Physiology) had participants interact face-to-face and then play economic games, so it seems relevant to the current study. There is also a literature based on the “fast friends” procedure; Sprecher 2021 (JSPR) might be relevant.

Authors reply to R1.5: Indeed, thank you for providing these references. We now acknowledge them in the main manuscript.

*p.5, lines 106-109: “While insightful, most of these studies, however, rely on non-interactive designs using static stimuli like photographs [16, 28], videos [29], or isolated voice recordings to study partner perceptions [17-18, 30] while only a small number of studies explore these phenomena in real-life social interactions [31-32].”*

6. In addition, there’s research on the inferences made from people’s voices and the effects of vocal parameters on social decisions. This would provide good theoretical grounding for analyzing the vocal parameters recorded from the conversation. For example, Aung et al., 2024 (Psychological Science) is a good, recent cross-cultural study of social perceptions based on voice pitch, and there’s a bunch of research on voice pitch affecting election outcomes (e.g. Tigue et al., 2012, EHB). Among hunter-gatherers, men with deeper voices are actually better hunters (Smith et al., 2017, Evolutionary Psychology), so there’s a plausible evolutionary account of why this would be relevant to partner choice.

Authors reply to R1.6: Thank you for providing these references, we acknowledged them in the manuscript, such as the literature that acknowledges the usefulness of social cues in navigating the social world. However, we wanted to avoid going too much in the evolutionary approach of understanding the relevance of these cues, as we were also citing literature from the perspective of social evaluations and social psychology, that was only interested in the effect of these cues, without explaining the function of the cues.

7. The passage on p.5 lines 104-111 could use a bit more explanation. It’s not clear immediately what you mean by manual annotation and automatic annotation.

Authors reply to R1.7: We clarified what was meant by manual and automatic annotation.

*p.5, lines 113-118:” Manual annotation, where annotators review behavioral data and label specific non-verbal behavioral cues, is both time-consuming and inadequate for capturing subtle nonverbal behaviors (e.g., acoustic behaviors) and hard to use for tracking their rapid changes in behavior. On the other hand, automatic annotation, using algorithms powered by machine learning (ML), to analyze and label behaviors, offers a more efficient way to capture a wide range of nonverbal cues [33].”*

8. The stuff about the actor-observer effect (e.g. p. 6 lines 126-128) seems like a bit of a stretch and not really necessary. People judging their conversation partner are still an observer with respect to their partner. This part of the framing, and testing the relationship between participants’ perceptions of their partners and the partners’ self-perception, was the least interesting to me. I’d suggest dropping it or maybe moving those results to SI – there’s plenty else of interest here that this experimental design can speak to more directly.

Authors reply to R1.8: Indeed, we removed this comparison from the manuscript, and we put the part of mis(alignment) of self- and other perceptions in the supplementary file.

*p.6. lines 131 – 133: “This distinction is important as it is well known that individuals who interact and observe the interaction have access to different information when forming perceptions [41].”*

9. On p. 10 lines 213-220, make it clearer that everything is happening online.
10. p. 16 lines 340-343: That’s a sloppy sentence. Please make it clearer.

Authors reply to R1.10: We made the sentence clearer.

See p.15, lines 338-348: *“Person Perceptions were assessed using established scales for warmth (comprising sociability and morality), competence [52], similarity, and physical attractiveness. Warmth was measured with a six-item scale: three items assessed sociability (e.g., "How sociable/friendly/kind is this person?"), and three items measured morality (e.g., "How trustworthy/honest/sincere is this person?"). All subscales demonstrated high internal consistency (Warmth: α_photo_ = .91, α_conv_ = .90; Sociability: α_photo_ = .81, α_conv_ = .83; Morality: α_photo_ = .91, α_conv_ = .90). Competence was assessed with items like "How intelligent/skillful/competent is this person?" (α_photo_ = .91; α_conv_ = .91). Physical attractiveness and similarity were measured with single items (e.g., "How physically attractive/similar in beliefs is this person?"). Perceptions were recorded on a 7-point Likert scale after viewing a photo and after each conversation.”*

11. p. 20 line 416-p. 22 line 460 should be moved to the Methods section.

Authors reply to R1.10: We moved the information about the preprocessing to the Method section.

12. p. 24 line 495: what do you mean by batch size?
13. p. 26 line 546: are those the reported statistics for an interaction effect? I think I figured it out from context, but it’d be better to be more explicit about what is being tested and reported in each result.
14. p. 27 line 580: By cooperative behavior, you mean amount sent in the trust task, right? I think little reminders like this would help the reader understand everything – there’s a lot going on here.
15. p. 28 line 586: significant predictor of what?
16. p. 28 line 589: “the role of partner selection in partner selection” – please proofread and fix.
17. p. 28 line 609-p. 29 line 610: I think you should make clear that the automatic annotations of facial and vocal cues could weakly predict perceptions of warmth. The vast majority of the variance in warmth perceptions was unaccounted for by the machine learning algorithms. It’s cool that automatic annotation predicted anything at all, but I think that some perspective regarding the effect size is necessary here.
18. p. 30 lines 636-650 discusses the cues that were and weren’t present in the conversation, but omits what the participants actually said. I think this should be mentioned as the likely explanation for most of the variance in warmth and competence perceptions, and the contents of the conversation would make a good target for future research. (Maybe expand on the mention of “verbal information” in line 664.)
19. p. 33 lines 709-710. It’s a bit strange that warmer partners were not more cooperative but selected partners were more cooperative, given that warmer partners were more likely to be selected. It seems like warmth promotes selection, and selection does predict cooperativeness, but the warmth ◊ cooperativeness relationship is not independently significant. I think perhaps this could be interpreted with a bit more nuance than just saying that partners perceived as warmer were not more cooperative.

Author replies to R1.12 – R1.19: Thank you for this detailed overview of the minor points. We addressed each of them.

Reviewer #2: This manuscript presents an exploration of person perception and partner choice. I’m embarrassed to say I don’t have much constructive to say here. The aims are clear, important, and target well-defined gaps in the literature. The methodology is inventive and impressive: I am impressed with the thorough data collection, the use of two behavioral tasks that separately isolate competence and warmth, as well as the effort to automatically annotate live interaction data—all with Prolific participants! I can see this study laying a useful foundation for future work on partner choice. The results are interesting and compelling: that perceptions of competence and warmth from real interactions independently predict partner choice for the distinct tasks is quite interesting.

My only slight criticism is that the aims of the manuscript feel a bit diffuse. It feels to me that there are two manuscripts here: one on partner choice and one on person perception. As a person admittedly more interested in the partner choice elements here, although technically very impressive, I don’t see as much value added by the automated annotation results. If we know that people are forming perceptions of warmth and competence from their interactions with their partners and we know that these perceptions are guiding their choice behaviors, what do I learn from knowing that a machine learning model can also predict some of the variance in some of the perceptions from the interaction data? This is, I concede, somewhat of an aesthetic choice: I’m sure a researcher interested more in person perception would find the annotation results and the perception accuracy results more interesting than the partner choice results. It seems to me that these could be two different manuscripts, with each set of results given the space and attention they deserve. Or one set of results could be backgrounded to supplementary material. However it is resolved, these distinct foci at present don’t really cohere into a single manuscript for me at the moment.

R2.1: We are glad that you liked the paper and thank you for sharing your thoughts. Indeed, we see how the part of the paper that about non-verbal cues and person perceptions is could potentially be a single paper. However, we wanted to include both topics in one paper, so we have the complete storyline of how these person perceptions are formed, what is the role of these perceptions (and task affordances) in partner selection, and lastly observing the consequences of person perceptions on cooperative behavior. Following the recommendation of the editor, we revised the discussion and introduction to make a more coherent story. Also, coming back to your question on the added importance of the machine learning pipeline, it is a very good point, and we realized that we did not highlight these issues properly in the discussion. Now, we highlighted this aspect in our discussion and explain what we think was the added benefit of using the machine learning approach. We hope these revisions further improve the coherence of the manuscript.

*p.29 lines 655 – 662: “Understanding whether machine learning can predict warmth and competence is important for two reasons. First, it provides new methodologies to model and analyze complex multivariate temporal processes between non-verbal cues of facial and acoustic behavior and social perceptions of warmth and competence. Second, it provides insights for fields like AI (Artificial Intelligence) in exploring whether models trained on non-verbal behaviors can predict abstract social phenomena, such as evaluations of potential partners. This could bring us closer to developing socially aware systems that assist in partner selection and promote cooperation [65-66].”*

*p.32. Lines 719– 730:* “Similarly, the usage of automatic annotation of nonverbal behaviors and the usage of machine learning to model social phenomena, such as person perceptions, have two implications. Firstly, automatic annotation was used as a less time-consuming alternative to manual annotation. Moreover, the combination of automatic annotation and machine learning modeling enables us to model complex multivariate temporal relationships and predict perceptions. This helps us to model more complex relationships, but it also highlights the potential of training models that can analyze non-verbal behavior and make predictions of important social phenomena during social interactions. This is important for current efforts in human-AI interaction. For instance, if we can create models that can automatically detect relevant human non-verbal behaviors and use them to reason about how individuals will be perceived in social interactions, we are a step closer to building AI systems that can use these reasons to provide feedback to humans and facilitate better social interactions [65-66, 70].”

On a very minor note: I think there may be an issue with the OSF page settings as it appears empty when I try to access it.

R2.2: Indeed, when the main project is opened it is empty, but when clicking on the pre-registration for the project, there should be a preregistration for the given dataset.
